# Supplementary material for: Impact of tourism on sustainable development in BRI countries: The moderating role of institutional quality
Source: PLoS One. 2022 Apr 18;17(4):e0263745. doi: 10.1371/journal.pone.0263745 (PMC9015125; doi:10.1371/journal.pone.0263745)
Supplement: S1 Appendix — (DOCX) [file pone.0263745.s001.docx]

Table 2.1: E-Government Ranking of Belt and Road Countries

| **Sr. #** | **Country** | **EGDI Ranking** | **Sr. #** | **Country** | **EGDI Ranking** |
| --- | --- | --- | --- | --- | --- |
| 1 | China |  | 33 | Lithuania |  |
| 2 | Mongolia MNG |  | 34 | Macedonia |  |
| 3 | Pakistan |  | 35 | Moldova |  |
| 4 | Bangladesh |  | 36 | Poland |  |
| 5 | Bhutan |  | 37 | Romania |  |
| 6 | India |  | 38 | Russian Federation |  |
| 7 | Nepal |  | 39 | Serbia |  |
| 8 | Sri Lanka |  | 40 | Slovakia Slovak Republic |  |
| 9 | Brunei |  | 41 | Slovenia |  |
| 10 | Cambodia |  | 42 | Turkey |  |
| 11 | Indonesia |  | 43 | Ukraine |  |
| 12 | Laos Lao PDR |  | 44 | Bahrain |  |
| 13 | Malaysia |  | 45 | Egypt |  |
| 14 | Myanmar |  | 46 | Israel |  |
| 15 | Philippines |  | 47 | Jordan |  |
| 16 | Singapore |  | 48 | Kuwait |  |
| 17 | Thailand |  | 49 | Lebanon |  |
| 18 | Vietnam |  | 50 | Oman |  |
| 19 | Kazakhstan |  | 51 | Qatar |  |
| 20 | Kyrgyzstan Kyrgyz Republic |  | 52 | Saudi Arabia |  |
| 21 | Tajikistan |  | 53 | Algeria |  |
| 22 | Albania |  | 54 | Burkina Faso |  |
| 23 | Armenia |  | 55 | Djibouti |  |
| 24 | Azerbaijan |  | 56 | Ethiopia |  |
| 25 | Belarus |  | 57 | Gambia, The |  |
| 26 | Bulgaria |  | 58 | Mali |  |
| 27 | Croatia |  | 59 | Mauritius |  |
| 28 | Czech Republic Czechia |  | 60 | Morocco |  |
| 29 | Estonia |  | 61 | Rwanda |  |
| 30 | Georgia |  | 62 | Senegal |  |
| 31 | Hungary |  | 63 | South Africa |  |
| 32 | Latvia |  | 64 | Kenya |  |
