# Supplementary material for: Impact of tourism on sustainable development in BRI countries: The moderating role of institutional quality
Source: PLoS One. 2022 Apr 18;17(4):e0263745. doi: 10.1371/journal.pone.0263745 (PMC9015125; doi:10.1371/journal.pone.0263745)
Supplement: Attachment — Submitted filename: Tourism-Response to Reviewers (1).docx [file pone.0263745.s003.docx]

**Ref: Decision on Manuscript**

**" Impact of Tourism on Sustainable Development in BRI Countries: The Moderating Role of Institutional Quality"**

**PLOS ONE**

We are very grateful to the ***Editor-in-Chief*** for giving us the opportunity to revise manuscript and resubmit our manuscript for consideration of publication in *PLOS ONE*. We are also extremely grateful for the valuable suggestions and constructive comments from the reviewers, which have been instrumental in strengthening the manuscript significantly. We have carefully addressed the reviewer each comment and do so sincerely hope that the manuscript meets the expectations. Please find improvements in file 'Revised Manuscript with Track Changes' in Yellow Highlighted texts. Moreover, after comments revision, overall proofreading was done by authors. Thank you very much for your precious time.

| **Sr. #** | **Comment** | **Response, Page, and Line Number** |
| --- | --- | --- |
|  | | |
|  | Please include the following items when submitting your revised manuscript:   - A rebuttal letter that responds to each point raised by the academic editor and reviewer(s). You should upload this letter as a separate file labeled 'Response to Reviewers'. - A marked-up copy of your manuscript that highlights changes made to the original version. You should upload this as a separate file labeled 'Revised Manuscript with Track Changes'. - An unmarked version of your revised paper without tracked changes. You should upload this as a separate file labeled 'Manuscript'. | Following items have been submitted as per PLOS One requirement. |
| **Reviewer: 1.** We are incredibly thankful for the valuable suggestions and constructive comments to improve the manuscript. We have carefully tried to address your comment and sincerely hope that the manuscript meets your expectations. | | |
| 1 | You can refer to the following articles to further improve the quality of your articles：  Impact of climate-related changes to the timing of autumn foliage colouration on tourism in Japan. Tourism Management, 70,262–272.  Impact of climate change on hiking: quantitative evidence through big data mining. Current Issues in Tourism. https://doi.org/10.1080/13683500.2020.1858037.  Effects of rural revitalization on rural tourism. Journal of Hospitality and Tourism Management (2021),https://doi.org/10.1016/j.jhtm.2021.02.008.  Study on the Impact of High-speed Railway Opening on China's Accessibility Pattern and Spatial Equality. Sustainability 2018,10,2943. doi:10.3390/su10082943.  The influence of high-speed rail on ice–snow tourism in northeastern China. Tourism Management（2020）, doi:10.1016/j.tourman.2019.104070. | Recommended articles have been cited in the manuscript to improve the quality of article.  Changes incorporated in “Introduction” section  Page 5, lines 1 to 9.  Previous researchers have investigated the factors that affect tourism, e.g. high-speed railway increases the approachability of ice-snow tourist destinations and contributing to the global tourism industry (Jin, Yang, Wang, & Liu, 2020; Yang, Guo, Li, & Huang, 2018). Rural revitalization promotes rural tourism activities and contributes to sustainable rural development in developing countries (Yang et al., 2021). While climate change is a critical factor that influences the tourism industry negatively. Humidity, sunshine duration and temperature affect hiking participation nonlinearly (Liu, Yang, Zhou, & Wang, 2020). Climate-related changes also impact tourism as it reduce the duration of leaf colouration in Japan, which causes a delay in autumn foliage colouration change (Liu, Cheng, Jiang, & Huang, 2019). |
| **Reviewer: 2.** We are incredibly thankful for the valuable suggestions and constructive comments to improve the manuscript. We have carefully try to address each comment and do so sincerely hope that the manuscript meets your expectations. | | |
| This study investigated the influence of tourism on sustainable development in BRI Countries, institutional quality as a moderating variable. The research has certain practical significance. But there are still some aspects to be improved. | | Thank you for the encouragement.We have carefully tried to address each comment and do sincerely hope that the manuscript meets your expectations. |
| 1. | There are too many contents of literature review. Try to select the most relevant articles to comment on the research topic of this paper, and there is no need to pursue comprehensive. | The literature review part is improved as suggested. We have tried our best to select most relevant articles. Word count of literature review has been reduced from 2039 to 1799.  Changes incorporated in “Literature Review” section.  Page 5 to 10. |
| 2. | As the key of this paper, the description of conceptual framework is insufficient. | Further description has been added on your kind suggestion.  Changes incorporated in “Research Mehtodology” section.  Page 12. |
| 3 | To what extent does tourism affect sustainable development should be clarified in the abstract? Otherwise, the impact of tourism on sustainable development seems to be obvious, even without this study. | Improved and incorporated the modification as suggested.  The current research findings have valuable contributions to academics as it offers novel insights about the 0. 351% influence of tourism on sustainable development at significance level of 1%, and it proposes valued suggestions to policymakers concerning tourism development strategies.  The outcomes of the two-step system GMM confirmed the statistically significant and positive dynamic nature of sustainable development and its relationship with tourism and other determinants at a significance level of 1% for BRI countries. Institutional quality enhanced the 4.693% sustainability path to achieve the sustainable development goals (SDGs) agenda with regionally interconnected countries at significance level of 1%. Renewable energy per capita and per capita income played a significant and positive role, while the exchange rate, household consumption, and urbanization negatively influenced sustainable development. |
| 4 | There is little description for Table 1, 2, 3, 4. Any table in the paper should be necessary, meaningful and clearly described. | Improved, further description has been added for Table 1,2,3,4.  Changes incorporated in “Results and Discussion” section.  Page 14 to 17.  **4.1 Descriptive Summary**  Table 1 exhibits the descriptive statistics in terms of the standard deviation, mean, minimum and maximum values, and observation count for the selected dependent, independent and control variables. Table 1 shows that a sample of 64 countries has 1024 observations from 2003 to 2018. The descriptive statistics show that all values are within the range.  Table 2 displays the variance inflation factor (VIF). We have calculated the VIF for independent variable tourism and control variables to confirm that there is no multicollinearity in our sample. If the value of VIF would be higher than five for any variable, then it indicates that a specific variable has multicollinearity issues (Mahmood, Han, Ali, Mubeen, & Shahzad, 2019). All VIF values in Table 2 are less than five, so our data is free from multicollinearity.  Table 3 displays the correlation between the variables. The results of table 3 depict that tourism, institutional quality (IQ), urbanization, renewable energy per capita (EQ), have a statistically positive and significant relationship, at 14.1%, 20.8%, 16.6%, and 30.9%, respectively, with sustainable development at 1% significance level. Per capita income is also positively correlated with sustainable development at 5% significance level. However, the exchange rate is negatively correlated with sustainable development at 5% significance level. The independent variable tourism interaction terms and the moderator institutional quality, i.e., Tourism*IQ, depict a positive relationship (12.9%) with sustainable development.  **4.2 Co-integration Test**  Co-integration of model panels was confirmed by the Westerlund test. The Westerlund test was performed to confirm the long-term association between the variables considered. The results of the Westerlund test have been depicted in Table 4, indicating a long-term co-integrating association between sustainable development and its determinants. This test specified that all panels were co-integrated. |
| 5 | Compared with previous studies, what is the innovation of this paper? Further clarification is required. | As mentioned in introduction part (Page#5) that previous study (Ibanescu et al., 2018) on this topic was done for one country only. Authors mentioned that data was poor because it does not allow contrasts to other states. This study has been done for 64 countries of BRI region. As most of countries of BRI region have natural beauty and economies of these countries depends on tourism. No previous study was done for this region.  Secondly, this study is the further extension of (Ullah, Pinglu, Ullah, & Hashmi, 2021a)’ work. Authors suggested the researchers to work on these variables in future. So, we measured the impact of tourism on sustainable development and considered institutional quality as a moderator. We have mentioned this study in our introduction part as a motivation for our paper.  Changes incorporated in “Introduction” section.  Page 5. |
| All new incorporated references cited in the manuscript. | | |
